# Neural Basis of Decision-Making and Assessment: Issues on Testability and Philosophical Relevance**

**DOI:** 10.4103/0973-1229.77441

**Published:** 2011

**Authors:** Gabriel José Corrê Mograbi

**Affiliations:** * *Professor of Philosophy of Science, Mind and Epistemology, Federal University Of Mato Grosso, Brazil.*; ** *Revised and peer reviewed version of a paper which won the Joshi-Bedekar College Award for Young Researchers at an International Seminar on Mind, Brain, and Consciousness, Thane College Campus, Thane, India, January 13–15, 2010.*

**Keywords:** *Decision-making*, *Inhibition*, *Attention*, *Self-control*, *Social Assessment*

## Abstract

Decision-making is an intricate subject in neuroscience. It is often argued that laboratorial research is not capable of dealing with the necessary complexity to study the issue. Whereas philosophers in general neglect the physiological features that constitute the main aspects of thought and behaviour, I advocate that cutting-edge neuroscientific experiments can offer us a framework to explain human behaviour in its relationship with will, self-control, inhibition, emotion and reasoning. It is my contention that self-control mechanisms can modulate more basic stimuli. Assuming the aforementioned standpoints, I show the physiological mechanisms underlying social assessment and decision-making. I also establish a difference between veridical and adaptive decision-making, useful to create experimental designs that can better mimic the complexity of our day-by-day decisions in more ecologically relevant laboratorial research. Moreover, I analyse some experiments in order to develop an epistemological reflection about the necessary neural mechanisms to social assessment and decision-making.

## Introduction

The discussion around the idea of decision-making will be oriented by the thesis that different levels of information processing in our brains interact. The more semantically loaded and interpreted character of frontal lobes’ information processing constitutes my epistemic credentials to sustain the argument that sheer determinism applied to living creatures, especially human beings, is as nonsensical as the metaphysical freedom of the will. I present empirical evidence on how higher-cognitive functions could control more basic stimuli and interpret that as the underlying necessary conditions to decision-making.

## Social Assessment

Cunningham and colleagues (Cunningham *et al*., 2004) promoted a study of the neural components of automatic and controlled social evaluation. The design of the experiment was basically the following: Black and White faces were shown to White participants while they were scanned using functional magnetic resonance imaging (fMRI). An extremely positive feature of this experiment: participants were not told they were taking part in a social evaluation experiment. All they knew was that they had to judge whether the image appeared on the left or right side of a fixation cross. Thus, no previously formed intention was generated by training or external cueing.

When the faces were presented in subliminal mode (30 milliseconds - “ms” from now on), activation in the amygdala was greater for Black than for White faces. Considering that the amygdala was a brain region associated with emotion that would be a representation of automatic fear or alertness, when the faces were presented in supraliminal mode (525 ms), this difference was particularly diminished. Accordingly, frontal cortex areas associated with control and regulation showed greater activation for Black than White faces.

Using Implicit Association Test (IAT), the authors were able to show that greater race bias correlated with greater difference in amygdala activation between Black and White faces. And what makes this correlation more precise is that the magnitude of amygdala activations was individually correlated to negative scoring in the IAT in each participant of the study. And that was made using precisely the same photos in both studies. The greater fontal activity was correlated by its turn with two tests that measure the intention to answer without prejudice: *Modern Racism Scale* and *Motivation to Respond Without Prejudice*. Again, it was possible to correlate the final numbers of those two tests with the magnitude of activation in the frontal lobes in each participant of the study individually. And the scoring in those two tests was directly proportional to frontal lobes’ activations.

Frontal activity generated a reduction in Black–White differences in amygdala activity from the 30 ms to the 525 ms condition. Beyond the above-cited correlation, Cunningham *et al*. (2004) showed, as well, that a direct comparison of amygdala activation for Black and White faces in the 30 ms and in the 525 ms conditions suggested an interaction between the amygdala and the frontal cortex. They showed that the Black–White difference in activation observed in the 30 ms condition was considerably reduced in the 525 ms condition, when automatic attitudes were supposedly controlled by more semantic loaded processing, F (1, 12) = 5.25, *P* < 0.05. As the authors stated: “These results provide evidence for neural distinctions between automatic and more controlled processing of social groups, and suggest that controlled processes may modulate automatic evaluation” (Cunningham *et al*., 2004).

The activities of certain areas of frontal cortex were increased for Black faces relative to White faces in the 525 ms condition (PFC; Brodmann’s Area, BA, 47), t (12) = 4.04, *P* < 0.005; right dorsolateral PFC (BA 9), t (12) = 4.88, *P* < 0.001; and anterior cingulate (BA 32), t (12) = 5.82, *P* < 0.001. The authors used the strategy of subtracting the magnitude of the Black–White difference in amygdala activation in the 525 ms condition from the corresponding difference in the 30 ms condition to generate an index of amygdala modulation by contrasting the results in both temporal conditions. The increasing frontal activity was proportional to the decreasing of amygdala activation. As the authors assert:

*These results, combined with previous investigations of intergroup attitudes, suggest that implicit negative associations to a social group may result in an automatic emotional response when encountering members of that group. Yet when participants had the opportunity to process Black and White faces for 525 ms (and reported seeing the faces), we observed activity differences not in the amygdala, but in areas of PFC (BA 47 and 9) and anterior cingulate (BA 32) - areas associated with inhibition, conflict, and control* (Cunnigham *et al*., 2004, p811).

## Why the Interest in This Experiment?

Obviously, the validity of this experiment for sociological purposes could not be extended to different socio-cultural contexts. Nevertheless, as we are not concerned with this sociological aspect of the experiment, these contextual limitations will not be addressed here. Our interest in this experiment is directly related to its approach to different levels of information processing, stressing the more automatic character of lower levels in comparison to the more controlled aspect of higher cognitive functions. And what is still more interesting is the possibility of control of more basic information processing by the higher-cognitive functions as is shown in the experiment:

*Moreover, a direct comparison of amygdala activation for Black compared with White faces in the short- and long-duration conditions resulted in a significant interaction. … the Black–White difference in activation observed in the 30-ms condition was significantly reduced in the 525-ms condition, when presumably automatic attitudes were counteracted by more positive controlled attitudes, F(1, 12)=5.25, *P* <.05. At the same time, areas of increased activity for Black faces relative to White faces in the 525-ms condition … were observed in right ventrolateral prefrontal cortex (PFC; Brodmann’s Area, BA, 47), t(12)=4.04, *P* <.005; right dorsolateral PFC (BA 9), t(12)=4.88, *P* <.001; and anterior cingulate (BA 32), t(12)=5.82, *P* <.001. We subtracted the magnitude of the Black–White difference in amygdala activation in the 525-ms condition from the corresponding difference in the 30-ms condition to generate an index of amygdala modulation. Correlations between this modulation index and participants’ Black–White contrast maps (voxel by voxel) for the 525-ms condition indicated that the modulation of amygdala activation was associated with increases in activation in dorsolateral PFC, t(12)=3.07, *P* <.005; MNI: x=33, y=48, z=36, and anterior cingulate, t(12)=2.98, *P* <.01; MNI: x=3, y=6, z=33* (Cunningham *et al*., 2004, p810).

They show that the modulation index and participants’ Black–White contrast maps (voxel by voxel) for the 525 ms condition could be directly and proportionally correlated with increases in activation in dorsolateral PFC and anterior cingulate, reinforcing the idea that frontal areas would be controlling the final behavioural output.

Comparing what is stated by the authors with my account of causal relevance of the mind, we find that bottom-up stimuli could start the information processing that is, by its turn, monitored by top-down control. We are not excluding the possibility of rational pre-planning to elicit future actions. Although, even if this heuristic reasoning would generate future goals, it must be instantiated in more basic information processing, namely bottom-up afferent stimuli and basic processing. Rational control does not come *ex nihilo* as dualists believe. The fashioning of rationality imputed to frontal lobes’ capabilities must be based on the circuitry and connectivity that integrates different levels of information processing. This higher-level organisation and its emergent properties arise from the relationship of individual neurons acting as a whole in neural groups and the connections between those neural groups. These results suggest that higher cognitive control functions would modulate the lower-level automatic evaluation. The authors are especially interested in the social character of these findings; nevertheless, my own interest in this experiment is only concerned with the possibility of control elicited by the more semantic loaded functions processed in the frontal lobes. That is exactly what I take for being emergent properties and its subtractive mechanisms responsible for control, suppression, repression, and selection.

My interest in this experiment is directly related to its approach to different levels of information processing, stressing the more automatic character of lower levels in comparison to the more controlled aspect of higher cognitive functions. The participants could not control the reactions when those perceptions were not consciously attended. But when they consciously attended, they could inhibit some of the potential improper behaviours. That is self-control. I have looked for similar or refutative work in the literature since this study was published, and not found any.

## Adaptive vs. Veridical Decision-Making

In 1999, Goldberg and Podell wrote a paper called “Adaptive versus Veridical Decision Making and the Frontal Lobes” (Goldberg and Podell, 1999). Veridical decision-making presupposes the idea that of one of the answers was the only correct one. Although the great majority of our choices were adaptive and did not have a unique transpersonal correct answer, adaptive decision-making was particularly dependent on the prefrontal lobes, differently from veridical decision-making. The ecological relevance of experiments dealing with adaptive decision-making was superior to those dealing with veridical decision-making:

*(…) the prefrontal cortex is central to the mechanisms of consciousness, owing to its unique role as the point of convergence of neural inputs from the organism’s external and internal milieus. The integration of inputs informing the organism about these two milieus is at the heart of adaptive behaviour and intentionality. The organism’s “best” response cannot be inferred from the properties of the external situation alone, since the choice of such a response depends on the organism’s needs and on how these needs are represented in the ’’working theater’’ of consciousness* (Goldberg and Podell, 1999, p364).

That is why the authors severely criticised the standard frontal lobe’s tasks as Wisconsin Card Sorting Test and Stroop Test. Thus, they designed Cognitive Bias Task (CBT) to promote a study that could take into consideration preferences of the people under study. The authors showed that patients with prefrontal lesions were more sensitive to the difficulties of CBT than to those present in the control experiment based on veridical decision-making. Comparing what is stated by the authors with my account of causal relevance of the mind, we find that bottom--up stimuli could start the information processing that is, by its turn, monitored by top-down control. Although this is a relatively old study, the next one sheds more light (see Arana *et al*., 2003).

## Your Favourite Food or Today’s Special?

Arana and colleagues (Arana *et al*., 2003) designed a very interesting experiment, one of the most ecologically relevant I have ever seen. The experiment focussed on appetitive incentive and decision-making between menus. The “restaurant task” was a highly adaptive paradigm. It could mimic very closely at least part of our assignment of values and the difficulty of choice in our day-by-day contexts. Unlike the great majority of the experiments trying to test decision-making, the one in discussion here really coped with competing possibilities of choice.

As food preference was extremely shaped by personal taste, the menus were especially tailored to each participant. Thus, the participants went under a questionnaire to establish their food preferences before the experiment. This measure allowed tailoring competing menus that could be rated in terms of incentive value.

The general conclusion in terms of functional areas involved shows that amygdala was especially related to process the incentive value, and the orbitofrontal cortex was particularly activated when participants had to decide each of the possible menus they would prefer to order.

The subjects took part in 12 positron emission tomography (PET) scans; in half of them high-incentive menus were presented and the other half were presented low-incentive menus. Low-incentive menus were defined as food that the subject would gladly eat but not as one of his/her favourites. In half of both low- and high-incentive values, subjects were requested to make a choice.

The amygdala activation was directly proportional to participants’ assignments of values. As in the current experiment people under study were satiated, we can infer that amygdala was strictly correlated with hedonic attribution of values. The medial orbitofrontal cortex was probably active even when a decision was not required because when we were faced with two competing stimuli, we were already assigning values to those items.

As decision-making is our main subject, let me focus specifically on the OFC activity during choice of competing stimuli:

*(…) a region of right lateral orbitofrontal cortex showed significantly increased activity specifically on trials involving choices between high-incentive menus. Subjects’ ratings of the menus demonstrated that choices between high incentive foods were more difficult to make than those between low-incentive foods; thus, when choosing between these foods subjects may have had to suppress responses to the other desirable items to select their most preferred item* (Arana *et al*., 2003, p9636-9637).

This is a very good hypothesis of how complex decision-making between high-incentive values is done. The election of one of the possibilities is given by the suppression of others. When we have two or more stimuli that offer very good reward prospects but we are supposed to select only one, we have to suppress the other(s) to pick up the most promising one.

Economic value is a central issue in decision-making and it is not considered here. That would imply that for the simplest case of our day-by-day menu choices, three different variables would be present: physiological values, hedonic values and rational economic choice values. And to study those variables in a sufficient way, we would have to establish the possible interactions of those systems, taking into consideration weights of the importance of each of them in different situations and in dissociable and comparable conditions. We have still not had an experiment by which all those variables in separable and comparable ways have been studied.

## Concluding Remarks [see also Figure 1]

**Figure 1:**
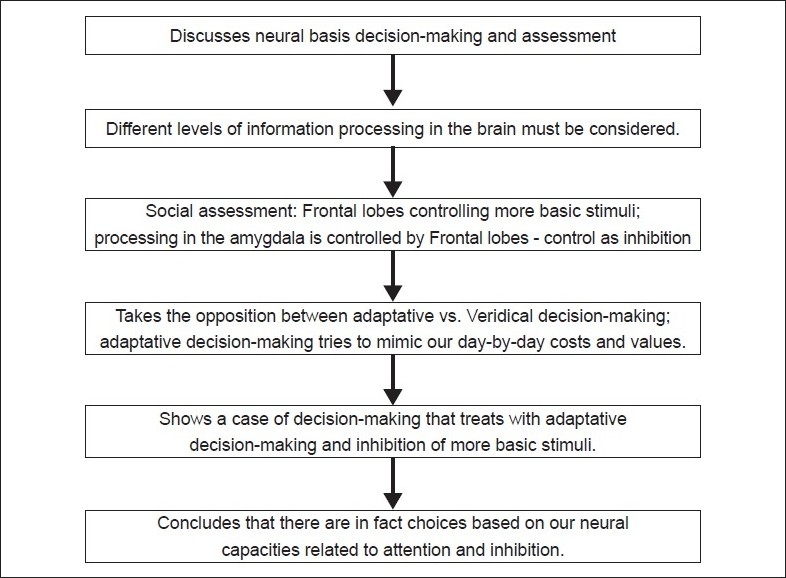
Flowchart of paper

Living beings are self-regulated. Even an amoeba in a petri’s plate, if you light up a flame on one of the plate sides, would escape to the other one. Thus, we are not stones that would explode or melt under the flame. Thus, fatalism is not the case. Even though we are not free in a metaphysical sense, attention and top-down control increase our possibility to decide among available and viable options. On one hand, it is the capability to control more basic stimuli and inhibit their undesired consequences, and on the other hand, it is our attention as a capability to maximize stimuli that constitute our power of choice.

### Take home message

Decision-making and assessment depends on inhibition of concurrent stimuli in favour of a given one. Inhibition is the physiological basis of self-control. Reasoning, as a higher-level type of information processing, can modulate more basic stimuli inhibiting concurrent stimuli in favour of a given one.

## Questions That This Paper Raises

1. Which are the laboratorial limits for testability in decison-making experiments?
2. Could we ever test decision-making perfectly mimicking the costs and values of our day-by-day real contexts?
3. Is inhibition the only strategy to choose?
4. Is top-down control always involved in decision-making?
5. Do we have free will?
6. To what extent do we have room for choice, if any?

## About the Author

Gabriel J. C. Mograbi is Professor of Epistemology, Philosophy of Mind and Science at the Federal University of Mato Grosso (UFMT) in Brazil. He had previous appointments with the Federal University of Rio de Janeiro (UFRJ) and the State University of Rio de Janeiro (UERJ). Gabriel Mograbi has BA, Master and PhD degrees in Philosophy from the Federal University of Rio de Janeiro (UFRJ). During his PhD, he was a Visiting Scholar at the University of California, Berkeley, where he worked under the supervision of John R. Searle. Gabriel Mograbi co-ordinates two research groups in epistemology of neuroscience. He has been presenting his works at the ASSC and TSC annual meetings and is a member of the former association. Gabriel Mograbi worked as an instructor/discussion leader for Professor’s Bernard Baars’ (Researcher at The Neuroscience Institute) Web course on Consciousness held by the University of Arizona.
